# Reconstructing historic and modern potato late blight outbreaks using text analytics

**DOI:** 10.1038/s41598-024-52870-2

**Published:** 2024-02-15

**Authors:** Ariel Saffer, Laura Tateosian, Amanda C. Saville, Yi-Peng Yang, Jean B. Ristaino

**Affiliations:** 1 Center for Geospatial Analytics, North Carolina State University, Raleigh, NC USA; 2 Department of Entomology and Plant Pathology, North Carolina State University, Raleigh, NC USA; 3 Emerging Plant Disease and Global Food Security Cluster, North Carolina State University, Raleigh, NC USA

**Keywords:** Plant sciences, Mathematics and computing

## Abstract

In 1843, a hitherto unknown plant pathogen entered the US and spread to potato fields in the northeast. By 1845, the pathogen had reached Ireland leading to devastating famine. Questions arose immediately about the source of the outbreaks and how the disease should be managed. The pathogen, now known as *Phytophthora infestans*, still continues to threaten food security globally. A wealth of untapped knowledge exists in both archival and modern documents, but is not readily available because the details are hidden in descriptive text. In this work, we (1) used text analytics of unstructured historical reports (1843–1845) to map US late blight outbreaks; (2) characterized theories on the source of the pathogen and remedies for control; and (3) created modern late blight intensity maps using Twitter feeds. The disease spread from 5 to 17 states and provinces in the US and Canada between 1843 and 1845. Crop losses, Andean sources of the pathogen, possible causes and potential treatments were discussed. Modern disease discussion on Twitter included near-global coverage and local disease observations. Topic modeling revealed general disease information, published research, and outbreak locations. The tools described will help researchers explore and map unstructured text to track and visualize pandemics.

## Introduction

In 1845, Ireland’s potato crop was destroyed by a plant disease. At the time, the germ theory of disease was not understood and the cause of the disease was unclear^1^. The pathogen destroyed potatoes and plagued Ireland for 7 years leading to mass starvation of the Irish people, emigration, and the Irish Potato Famine^2^. However, the first reports of the disease occurred in the United States in 1843 around the ports of Philadelphia and New York^3,4^. The plant disease pandemic spread over a three year period to more states and provinces in the northeast US and Canada and by 1845 was reported in Europe, the UK and Ireland.

Many theories were proposed at the time to explain the pandemic including a curse from God, bad weather, the *laziness of the Irish*, or a minute fungus^5–7^. M. J. Berkeley would ultimately elucidate the cause and named the fungus-like pathogen *Botrytis infestans*. Later, Anton DeBary would publish the complete life cycle and rename the pathogen *Phytophthora infestans*^8,9^. Initially, the center of origin of the disease and source of the nineteenth century outbreaks was suspected to be from South America^5,6^. Later, competing Competing theories suggest either a Mexican source^10–13^ or an Andean source^14–17^. A hybrid theory suggesting Mexico as the center of origin of the disease but the Andean region as the source of the outbreak strain has also been suggested by several authors^3,6,18^. Historic herbarium specimens have been used to identify and track the outbreak strain^19–24^ and genomic sequence data support an Andean source^22^.

The presence of the disease was recorded extensively in nineteenth century newspapers, letters, and government reports, many of which were documented in the annual US Commissioner of Patent Reports^25–27^. Prior to the formation of the US Department of Agriculture, agricultural reports including crop status and yield information were collected in the Patent Office reports. These documents included descriptions of location information as growers reported symptoms on their farms and chronicled their attempts at controlling the disease. The content also included excerpts from agricultural newspapers. These reports represent a valuable resource of information to better inform our understanding of the source of this important plant disease during the first outbreaks in the early days of the plant disease pandemic in the US prior to its spread to Europe and Ireland. Previously published maps have only inferred the general location and direction of spread of the disease over time on a broad scope and based on manual examination of the texts^4–6^.

Given the narrative nature of the patent reports and the sheer volume of information (over 6000 pages between 1841 and 1850), we used data analytics and natural language processing (NLP) to efficiently extract relevant location information and form a more complete visualization of the spread of this important plant disease pandemic that still threatens food security for millions in the developing world^17,28,29^. Late blight continues to be a disease of global concern particularly to small holder farmers that lack access to fungicides^17^.

While archival documents can provide valuable insights into the past, contemporary digital texts offer abundant and low latency information to track the current spread of pests. Modern pathogen observation and distribution data are typically collated directly from field observations, published databases such as USABlight.org^17,30^ and peer-reviewed journal articles. These sources provide useful information, but the data may be limited in spatial extent due to collection costs or there may be spatial bias based on where funding for studies is available^31^. Critically, there may be a delay between when a pathogen establishes and when it is documented and reported officially in peer-reviewed literature or public databases^31,32^. Recently available “big data” streams from multiple structured (e.g., published papers, archival printed documents, PubMed and CAB Abstracts, Google Ngram) and unstructured data sources (web news, image posts, Twitter feeds) have been used to monitor and map infectious diseases and invasive pests and set baseline information on first reports^33–36^.

We developed a workflow from historical and contemporary texts to extract relevant passages with mentioned places names and derive geospatial points for digital mapping and analysis (Supplementary Fig. 1). We analyzed the US Commissioner of Patent reports from 1843 to 1845 with the intent of locating reports that indicate the presence of potato late blight and connecting them to specific locations^25–27^. To obtain a broader view of contemporary literature and to provide context for the time period examined, we also analyzed the Google Books Corpus^37^, which includes millions of books written over 100 of years. Finally, we used Twitter posts from 2012 to 2022 to learn more about modern mentions and distribution of the disease.

Our overall goal was to use text analytic and data mining tools to track historic and modern late blight outbreaks and understand theories of the cause of the disease, origin and mitigation strategies. Our specific objectives were to: (1) use text analytics of unstructured historical reports from the US Commissioner of Patents Office to map historical (1843–1845) US potato blight outbreaks; (2) characterize theories on the source and spread of the pathogen and remedies for control; and (3) create modern late blight intensity maps using Twitter feeds.

## Results

A glossary of text analytics terms that readers can reference as the results and methods are discussed is provided (Supplementary Table 1).

### Impact of late blight in the US

**Figure 1:**
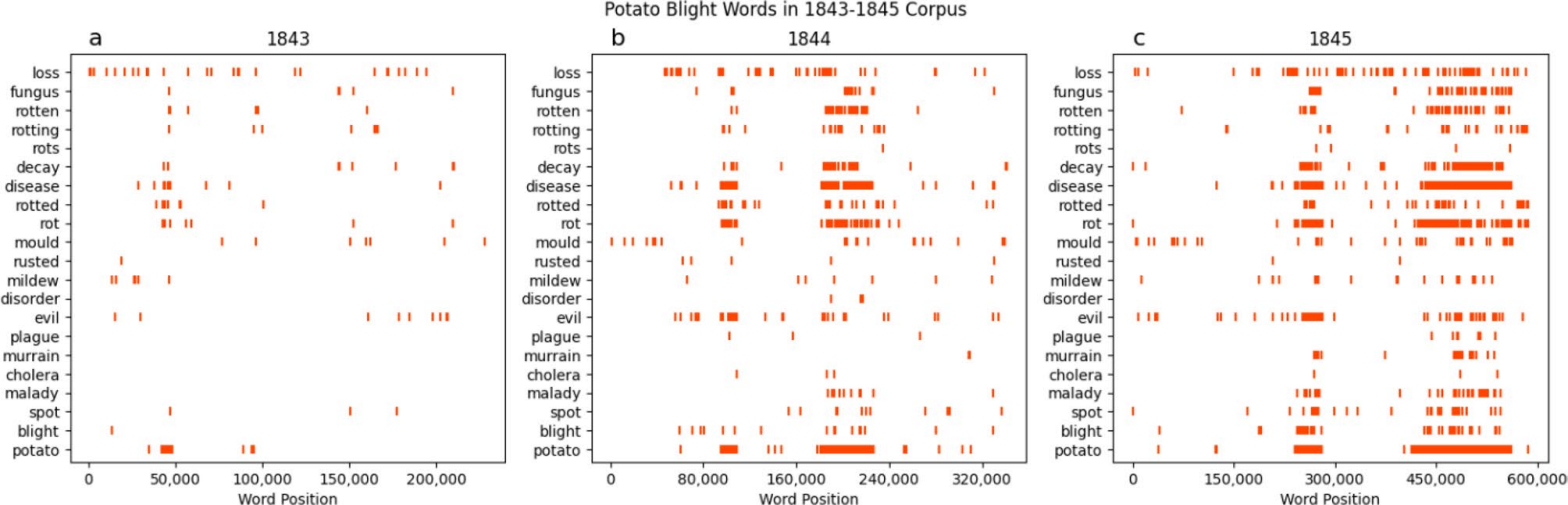
Lexical dispersion plots of word frequencies for different search terms indicated for potato disease from the US Commissioner of Patent Reports from (**a**) 1843 (**b**)**,** 1844 and (**c**) 1845.

We conducted an analysis of the frequency of key words and phrases relating to potato blight in the US Annual Commissioner of Patent Reports^25–27^. Lexical dispersion plots of data retrieved from the 1843 to 1845 reports revealed an increase in the frequency of words like “rot”, “blight”, and “disease” (Fig. 1**)**. These keywords occurred in the text in close proximity to the keyword “potato”, starting in 1844, suggesting an increase in the occurrence of the disease from the previous year. The frequency change also implies an increase in discussion about the disease in the reports as growers and scientists became aware of the new disease affecting potato crops and discussed the disease at local agricultural society meetings.

**Figure 2:**
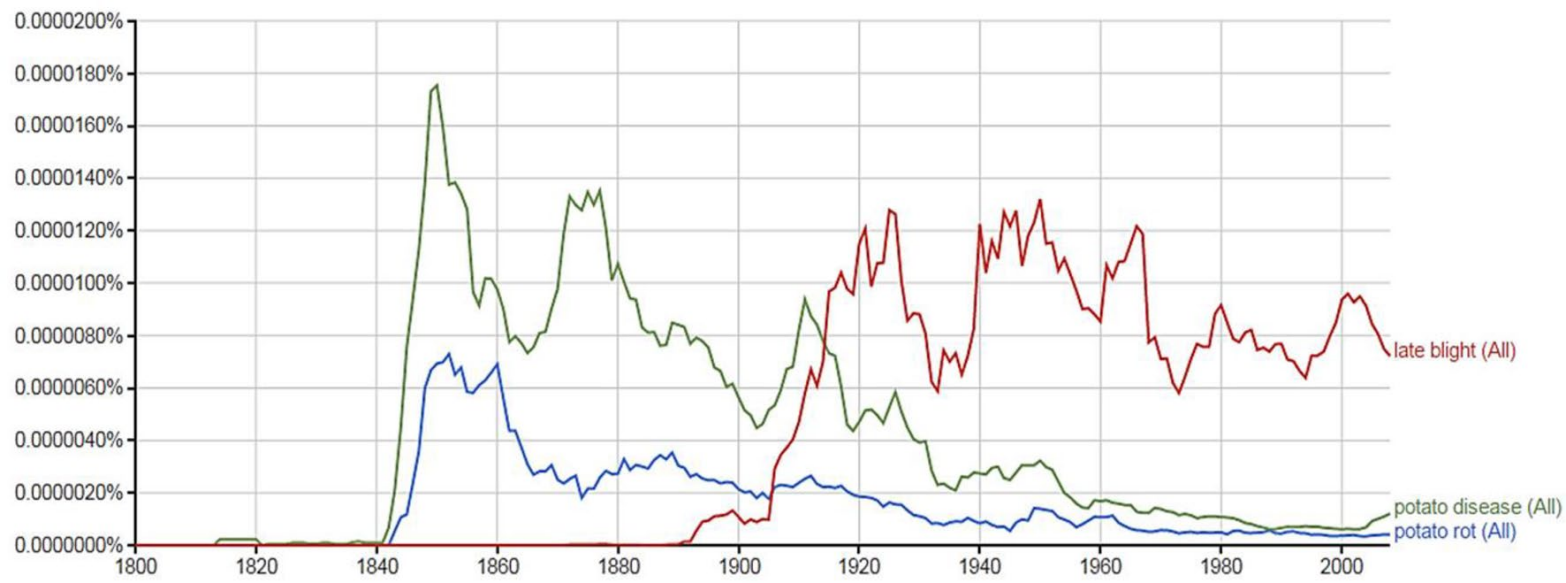
Google Ngram plot of three 2-g keywords searches for “potato disease”, “potato rot” and “late blight” in publications from the English corpus 1800–2019.

We also examined the reports of the potato disease with Google Ngram which included a broader look at literature from the time period. A Google Ngram search of the Google Books Corpus for three 2-g (“potato disease”, “potato rot”, “late blight”) revealed a spike in the occurrence of “potato disease” and “potato rot” around the mid-1840s that coincided with the Irish Famine outbreaks (Fig. 2). A second spike for “potato disease” was noted around the 1870s, which correlated with a second major outbreak of late blight that was recorded in historic literature^38^. A third spike, almost as large as the 1840 spike, occurred in the 1940s during the time period of World War II. The term “late blight” did not appear in the word corpus until around 1900, possibly in conjunction with both the observation of disease occurrence in late maturing varieties and the start of breeding efforts to produce blight-resistant potatoes that had delayed disease until “late” in the season.

**Figure 3:**
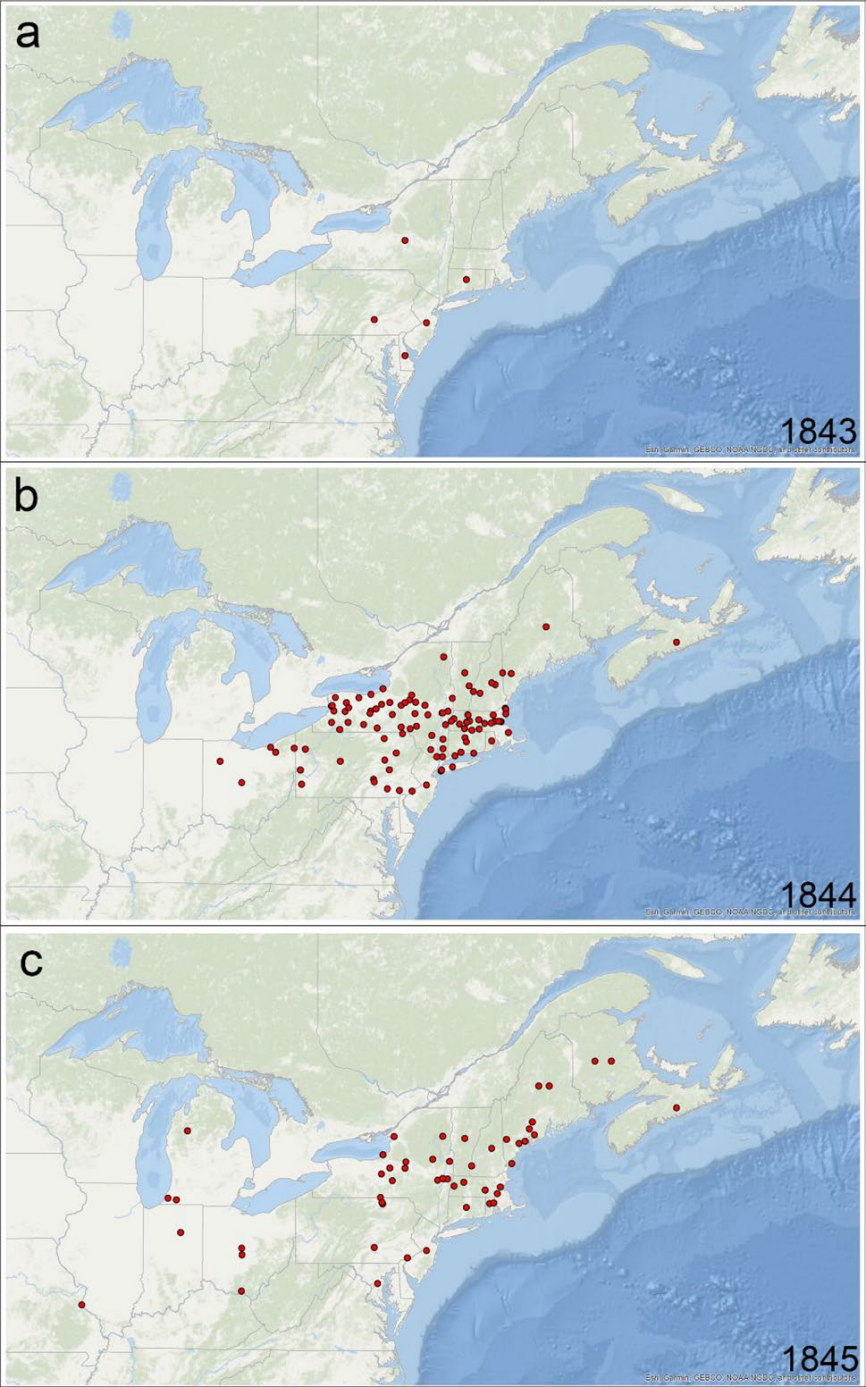
Maps of the geographic locations (occurrences) of the potato disease in the northeastern US and southeastern Canada from (**a**) 1843, (**b**) 1844 and (**c**) 1845 drawn from text analytics of US Commissioner of Patent Reports, 1843–1845. The data were mapped with ArcMap 10.8, ESRI, Redlands, CA (https://desktop.arcgis.com/en/arcmap/latest/get-started/setup/arcgis-desktop-system-requirements.htm).

Through keyword searches, we located sections of interest within the patent reports, which could then be examined in relation to theories on cause and source of the disease and means of control (Table 1). The letters and reports reveal the concern and alarm within the US agricultural community in 1844 and 1845 as it becomes clear that the disease observed in 1843 was not an isolated incident but increased in spread and occurrence with time and became a pandemic (Fig. 3). Terms used to describe the disease included “evil”, “murrain” and “plague” indicating the serious nature of the problem (Fig. 1). The Massachusetts Ploughman agricultural newspaper is documented in the 1844 report as stating “*Have you found out the cause of the strange rot in the potato crop? This is the anxious inquiry of almost every farmer we converse with.*” Numerous accounts of the struggles of scientists and farmers to understand the cause of the disease and potential treatments were also identified, chronicling the debate over the major theories of the cause of the disease that included insects, poor nutrition, bad weather, worn out varieties and seed of potato, or a minute fungus. The work of J. E. Teschemacher, an amateur botanist, is reported in detail in the 1844 reports including the symptoms of the disease on potato, microscopic observations and transmission of the “fungus’ (Table 1)^26^. Teschemacher subsequently published this report in the Gardeners Chronicles^39^.

**Table 1:** Specific quotes related to the cause, source and means of treating the nineteenth century potato disease.

*“many crops were worthless when dug from the ground; and almost all crops began to decay immediately after drying, and rapidly decayed till they were an extremely offensive putrid mass*”		
Subjects	Topics	Quotes
Causes of disease	Worn out varieties	The better opinion in those countries is, that by long propagation from the tuber, without recurring to the natural seed of the plant, it has lost a portion of its vital power, and hence is extremely prone to blight, rust, and to rot
	Fungus	Mr. Teschemacher, of Boston, tried, by microscopic examination, to find out the cause. He discovered in the potato a growth of fungus, which is a plant analogous to the mushroom family. These hung in seeds are invisible to the naked eye; they are readily carried about by winds, and will penetrate wherever air will. Being once introduced from Europe, their extensive dissemination here is very easy. These seeds falling on the potato, in favorable circumstances as to moisture, & etc., cause the disease
	Weather	Several theories have formerly prevailed on this subject. but, so far as we are acquainted, the most generally received one at the present time is, that a peculiar state of the atmosphere occasions the bursting of the sap vessels of the plant, by which the sap is exuded to the outside of the stalk and leaf. The sap, then, becoming acrid, together with the derangements in the functions of the plant, is supposed to occasion the blight or rust in grain
Crop loss estimates	33–50% or greater in some states	The loss last season from rot exceeded $700,000, and we fear that this year it will fall but little below $1,000,000. In no case have we heard of an increased crop, but the language, as applied to different sections, is" nearly 50 per cent less, owing to a rot which seized them before the time for taking them out of the ground
Methods of control	Lime, drying tubers in sand, ash	In 1843, after being put into the cellar; and so he picked out those that were affected, and put half a peck of soaked lime to each layer of the others, and they kept well. In planting, last spring, he put a table spoonful of lime in each hill; and after they were up, and before hilling, he applied to each hill about a will of a mixture of lime 2 bushels, plaster 3, and ashes 8. He had not one rotten potato
	Salt	In Pennsylvania, it (rot) prevailed in the moist mountainous region. A friend of his used about a half teaspoon of salt to a hill of potatoes when they began to set to prevent injury from worms:, and they were excellent where salt was used, the others not worth harvesting
	Manure	Towards the southern boundary, also, the report is: "Greatly injured by the rot; all of one-third of the crop was destroyed They suffered most on manured lands; on lands not manured, they escaped the disease entirely."
Geography		In the central western part of the state, on the Connecticut river, the potatoes were early struck with rust, and nearly one-third of the crop has rotted." ..for 1844, already given, that the potato rot or disease (as it is called) has extended and prevailed far more than in the year previous. So great has been the evil, that it excites serious apprehensions, unless something may be found to prove an effectual remedy
Sources of infected seed potatoes	Nova Scotia	The farmers of Nova Scotia, who shipped large quantities of this root to Boston during the past season, have long been acquainted with the disease, and call it the rot. It seems to pervade particular farms; and sometimes appears in the stalk, like rust, long before the potato has arrived at maturity; and, on cutting open the young root, the disease will be found to exhibit itself as black spots throughout the inside of it
	French potatoes	French potatoes, received three weeks before directly from Harve….on 3^rd^ of October, ploughed and all perfectly sound with the exception of pink eyed kidneys and French potatoes, which were entirely rotten
Origin of the disease	Bogota Andean source	The malady is very common on the table land of Bogota; that it is destructed in wet seasons, or even every year in damp spots
Variety differences	Some varieties more susceptible	The Chenango variety, which has been cultivated by farmers for a long series of years, has been most affected by the disease. The English whites and long reds have not suffered so much, because they have less constitutional defects; but these 'have for some years shown strong symptoms of decay in intelligent farmer of our acquaintance corroborates the opinion advanced in the above paragraphs, attributing the disease to the constant re planting of the same seed

Keyword searches also revealed text passages in which farmers were actively sharing information and developing methods to control the disease, including the use of lime, ash, salt, bluestone copper treatment of tubers, tobacco juice to ward off insects, and application of sulfur (Table 1). It was often mentioned that manured land had greater disease than non-manured land. Since tubers were rotting in the ground, many mentions of methods to store potatoes and keep them dry over the winter are recorded in the reports. The text analytics also documented how the disease affected different varieties of potatoes. A writer to the Bangor Whig newspaper indicated in the 1844 report that “…*the Chenango variety, which has been cultivated by farmers for a long series of years, has been the most affected by the disease while The English whites and long reds have not suffered so much*”.

Examination of name locations identified in close proximity to disease keywords also revealed potential sources of introduction of the disease, based on local accounts. A writer to the Bangor Whig newspaper indicated in the 1844 report that “*The farmers of Nova Scotia, who shipped large quantities of this root to Boston during the past season, have long been acquainted with the disease, and call it the rot*” (Table 1). This passage suggests that the pathogen may have been present in North America earlier than 1843 and that possible movement of the pathogen into the US could have occurred via Canadian potatoes (Table 1). There is also evidence suggesting that growers were obtaining new seed potatoes from South America and from Europe, due to a dry rot that was affecting potato tuber quality. There is mention of the disease having been present in South America in the 1845 report^27^, in which it is documented based on a letter from Joachim Acosta that “…*the malady is very common on the table land of Bogota, Columbia; that it is destructive in wet seasons, or even every year in damp spots*” (Table 1).

### Spread of late blight in the US, 1843–1845

We used geographic locations extracted from the patent report text to generate maps of the spread of late blight in the US and southeastern Canada from 1843 to 1845. These maps represent, to our knowledge, the most accurate spatial reconstruction to date of the movement of the pathogen from the first known reports in the US in 1843 (Fig. 3). Five unique locations in different states where the potato disease was present were identified in 1843, with accounts limited to states around New York and surrounding regions, including Pennsylvania, New Jersey, Delaware, and Connecticut (Fig. 3a). In 1844, the number of unique locations identified increased to 107 and the pandemic expanded to include six other states including Ohio, Massachusetts, Rhode Island, Vermont, New Hampshire, and Maine as well as the Canadian province of Nova Scotia (Fig. 3b). There were fewer new reports in 1845, with only 53 unique locations identified, but the number of states reporting disease continued to expand, including Michigan, Illinois, Indiana, Maryland, and the Canadian province of New Brunswick (Fig. 3c). Over time reports increase in frequency in the major potato growing regions and the surrounding northeastern US states. Mention is made of disease occurrence near rivers (Delaware, Connecticut rivers) which were often used to transport seed tubers into rural areas. Interestingly, the disease is still present in many of these same northeastern states as the maps of cumulative data from the USABlight.org reporting system indicates^17,30^.

### Modern day records of disease recorded from Twitter

**Figure 4:**
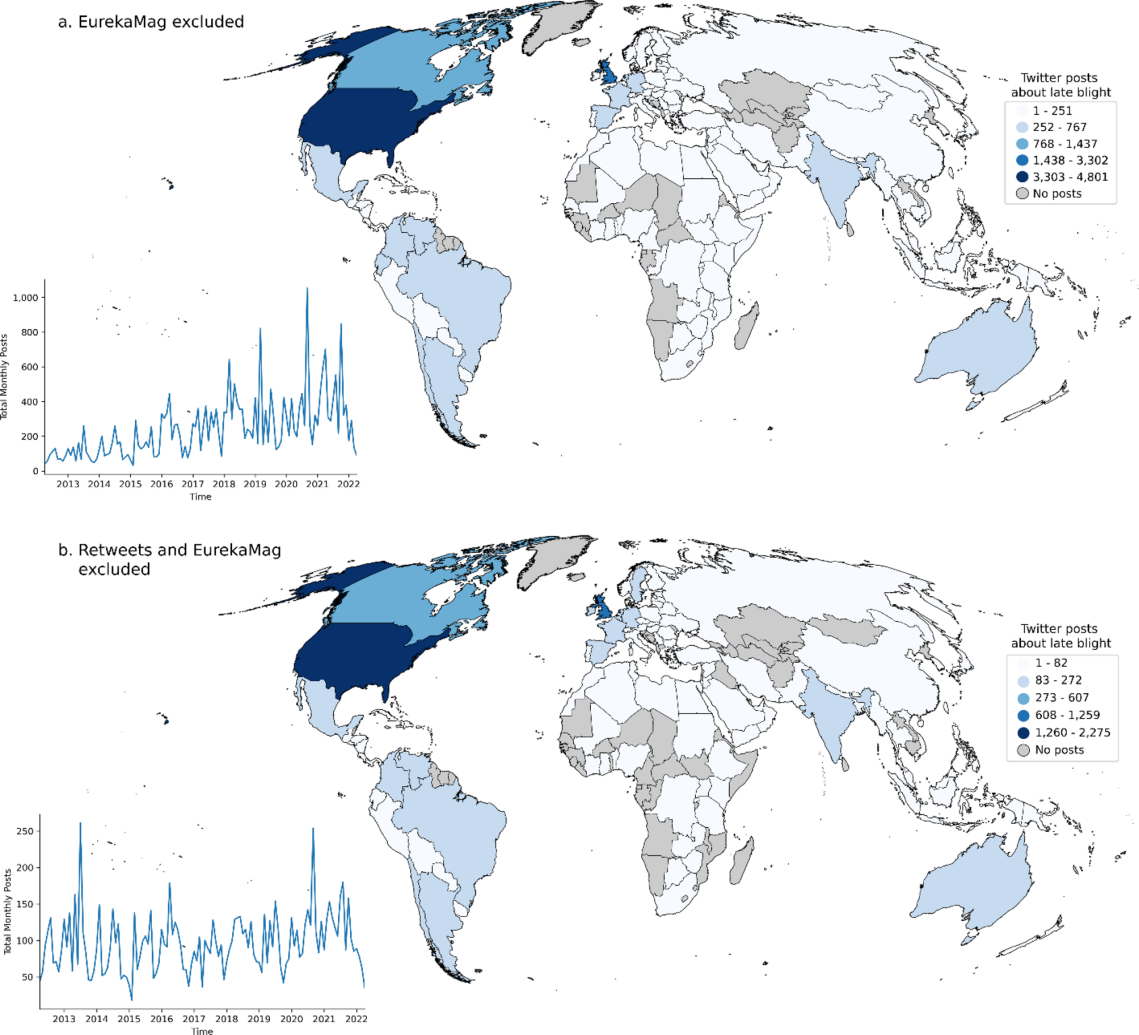
Global distribution map of 10 years (2012–2022) of Twitter mentions of late blight and related terms (tomato blight, potato blight, tuber rot, foliar blight, and variations of descriptions of *Phytophthora infestans.* (**a**) EurekaMag posts excluded and Retweets included. Insert shows peak velocity of Tweets occurred in September 2020; (**b**) EurekaMag and Retweets excluded. Insert (bottom left) shows that peak volume of Tweets occurred in July 2013. The maps in Fig. 4 were created using the geopandas package (version 0.12.2, https://geopandas.org/en/stable/) in Python 3.10.9. The World Countries Generalized shapefile was obtained from ESRI (https://hub.arcgis.com/datasets/esri::world-countries-generalized/about, and used to create this map.

We obtained 10 years of recent global Twitter posts mentioning the pathogen with a total of 41,720 posts in 39 languages (81% English), 22,879 with geographic locations from 132 countries (Fig. 4a). Excluding Retweets and posts attributed to a single scholarly literature delivery service (EurekaMag) reduced this number to 11,570 posts. 7615 of these posts contained geographic location names. The mentioned locations included places from 107 distinct countries (Fig. 4b). Most topics produced with topic modeling described research advances (e.g., “potato disease potatoes resistant farmers crops help crop could new”, “effector rxlr host plant potato targets via avr3a immune effectors”) and general information about the disease (e.g., “potato Irish caused disease famine million crop cause blight Ireland”). However, a single frequent topic in 6.26% of posts identified potential disease occurrence reports. The topic modeling represented this topic by its 10 most probable words: “tomato potato found confirmed rt county growers disease strain reported”.

Further targeting this topic, we extracted example posts that included descriptions of late blight presence and the emergence of particular strains at specifically named locations (e.g., “*Late blight found on potato in Adams County in central Wisconsin. Testing carried out by UW confirmed the strain was US-23*.”) and used this topic to train supervised machine learning classification. Unique Tweets in all languages were translated to English and 285 were labeled (late blight report, yes/no) by authors. Text classification identified 1931 relevant posts that include reports of *P. infestans*.

The geographic origin of the post is derived from a geolocation when provided by the user (0.05% of late blight posts) but for the remaining posts, we inferred this information from the user's profile or other heuristics at coarser geographic scales (i.e., state, country)^40^. However, the text content of posts contained additional, different, and more precise, geographic information useful in describing where an outbreak was occurring^41,42^. To this end, we applied a geoparsing pipeline using named entity recognition (NER) to extract locations from text classified as reports and using disambiguation to assign coordinates to the extracted locations.

**Figure 5:**
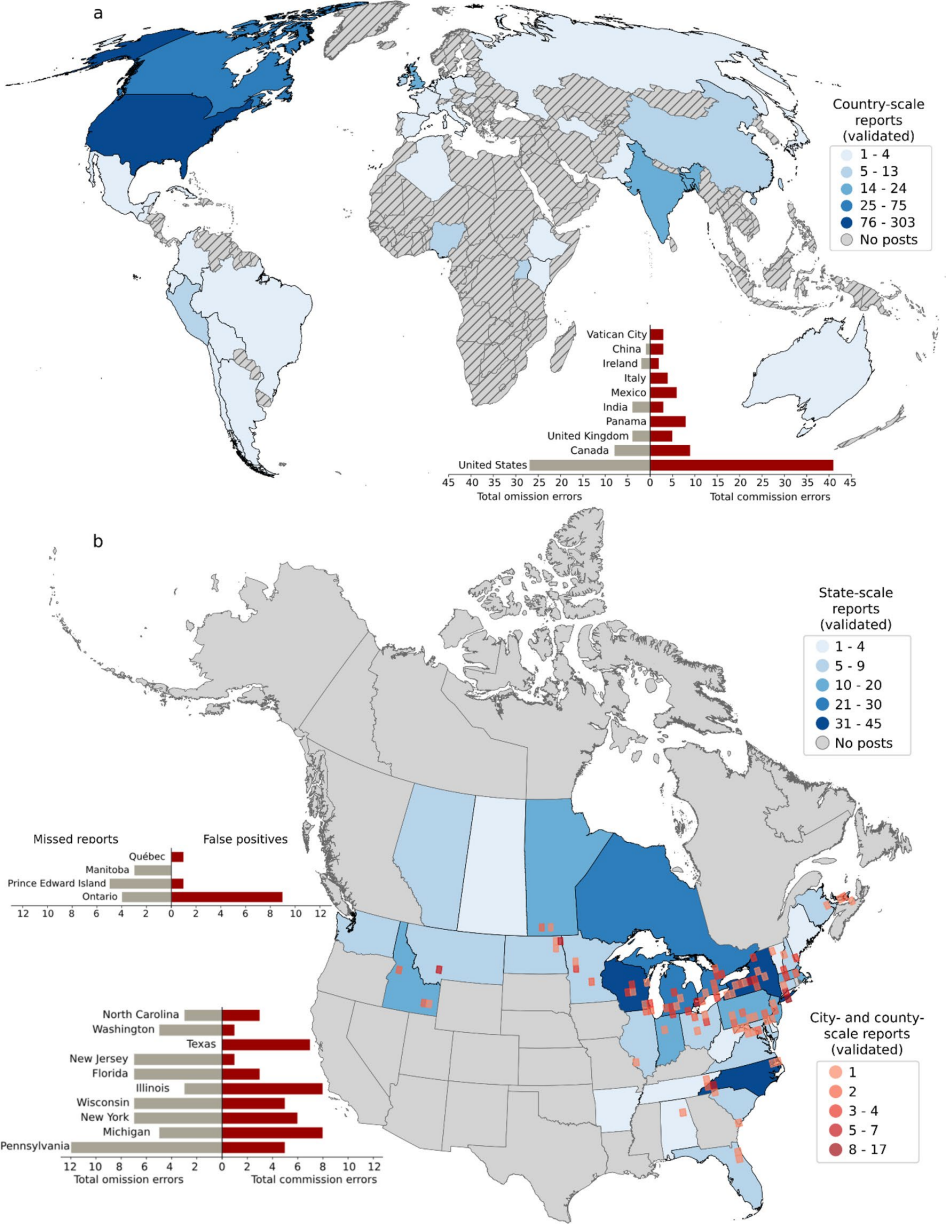
Late blight reports extracted and manually validated from Tweets (2012–2022) using supervised text classification and Named Entity Recognition with geocoding. (**a**) Number of times late blight was reported in posts globally, aggregated to the country scale. (**b**) The number of times late blight was reported in posts for the United States and Canada, aggregated to the state scale (shown in blue). Counts of location mentions at the city scale are aggregated to a 60 × 60 km grid (shown in red). Bar charts in both figures show the counts of omission (missed reports) and commission (false reports) errors identified during data validation for countries **(a**) and states (**b**) with the most errors. The maps in Fig. 5 were created using the geopandas package (version 0.12.2, https://geopandas.org/en/stable/) in Python 3.10.9. The World Countries Generalized shapefile was obtained from ESRI (https://hub.arcgis.com/datasets/esri::world-countries-generalized/about), and used for (**a**). The North America Political Boundaries shapefile from the Commission for Environmental Cooperation (CEC) (http://www.cec.org/north-american-environmental-atlas/political-boundaries-2021/) was used in (**b**).

Automatically geolocating place names is complicated by the fact that many place names are used to refer to multiple distinct locations. Disambiguation aims to correctly select the true location being discussed. We considered three ways to impute the context of posts to disambiguate locations: using no additional context, the country of origin as context, or additional locations mentioned nearby in text as context (see detailed approach in Methods). The NER analysis yielded 629 reports that included place name entities. Our disambiguation approach resulted in 575 posts including 602 geolocations at the country scale, with 429 posts mentioning 456 locations identified at the state scale, and 272 posts mentioning 283 locations identified at the city- or county-scale. Manual evaluation of positive reports identified 55 false positive reports (8.7% error rate for classification), 171 mis-identified places (21.1% error rate for geoparsing) and 52 additional missed places in posts. After correction, the United States accounted for 55.9% of places reported in posts, followed by Canada (13.8%), the United Kingdom (4.4%), India (4.2%), Bangladesh (3.5%), Nigeria (2.4%), China (2.0%), Ireland (1.5%), Peru (1.3%) and Uganda (1.1%) (Fig. 5A). In the contiguous United States and Canada, there were 395 validated reports from posts mentioning states and 201 with city or county-scale locations (Fig. 5B) when cities and counties were aggregated to 60 × 60 km grid cells by centroid location. We aggregated omission and commission errors in the automatically classified data by country and state (Fig. 5A,B, respectively). The locations reported provide a new supplementary dataset of spatiotemporal disease observations.

## Discussion

The text analytics of US Commissioner of Patent Office Reports revealed that the potato disease was first reported in the US in 1843 in a five-state area. The pathogen and subsequent disease spread rapidly in potato fields and by 1844 had expanded to the neighboring states in the northeast. In 1845, first reports of the disease into Europe, Scotland, and Ireland occurred, resulting in the Irish diaspora, mass starvation, death, and emigration of people from Ireland. Since the fungus-like plant pathogen was unknown to science at the time, the cause and source of the disease as well as remedies for control were widely discussed in agricultural reports, newspapers, and government pamphlets. In this work we have created the first accurate maps of the 1843–1845 outbreaks of potato late blight in the US. Our text analytics has also revealed information on theories of the cause and methods of control.

We developed a workflow for digitalizing historical documents, extracting information of interest and spatial data, and visualizing them on an interactive map for verification and analysis (Supplemental Fig. 1). The methodology accelerates the process of exploring voluminous unstructured text for the purposes of tracking disease observations over time and space based on key words and phrases. This method can be scaled to trace the movement of historic outbreaks of other diseases where historic text documents are available. We visualized the spread of late blight at a level of detail not previously used, and demonstrated the spread of disease from a five state area (New York, Delaware, Massachusetts, New Jersey and Pennsylvania) to the rest of the northeastern US and Canada.

In addition to geographic information, the targeted searches allowed us to locate passages of interest in the agricultural papers and meetings of agricultural societies of the time that described the efforts of the agricultural community, landowners, and amateur scientists to understand the agent that caused the potato disease and manage its consequences^26^. The observations on the “fungus” causing the disease first described in the Patent Office reports by Teschemacher and later by him in The Gardeners Chronicles^39^ preceded work done by Louis Pasteur to define the germ theory of disease^43^ and before the potato blight pathogen was officially identified and named by Berkeley^5^. These passages give us better insight into the contemporaneous ideas on the fungal nature of disease. Reports widely discuss the potato fungus as a consequence rather than cause of the disease, as the contagion nature of a plant pathogen was yet to be accepted^5,7^. Over time, the work of DeBary conclusively documented the life cycle of the pathogen^6,8,9^.

Due to the nature of the source material, we also observed mentions of grower anxiety and frustration as they contended with this new threat to their potato crops, summed up quite succinctly by Dr. Saml L. Dana in a letter from the 1844 Patent Office report^26^ in which he stated, “*I want light.*”. Terms were repeatedly used to describe the disease such as evil, murrain, cholera, and plague, reflecting some understanding of the contagion nature of the disease, but also the hopelessness of not having a clear answer as to the cause or prevention of further disease. Methods and strategies used for disease management and those that showed promise of success were often mentioned. The onset of the first potato blight outbreaks in the US and Europe led to the nascent formation of chemical control strategies to manage plant diseases. Growers used materials at hand on farms including lime, sulfur, bluestone copper steeps of seed potatoes, and salt, and many experimental trials are described in the reports (Table 1).

It is also clear from the reports that infected potato seed tubers were suspected as a source of the disease. The mention of seed tuber sources being imported from outside the US including from France, South America, and Nova Scotia is mentioned. There was a booming bat guano trade of Peruvian bat guano as fertilizer in the 1840s and shipments of potato tubers on those same steamships in days rather than weeks from South America would have enabled the pathogen to survive and enter US ports^3,4,6^.

The timing of the emergence of several distinct global potato blight disease pandemics over a 150-year time period was also confirmed through the use of keyword searches in Google Ngram with the larger corpus of source information. We used 2-g word (“potato rot”, “potato disease” and “late blight”) associated with the disease to search the broader literature. Google Ngram plots of late blight, potato blight and potato rot mentions in published literature revealed the 1845 peak in reports of the potato disease associated with the European and Irish outbreaks and a smaller peak in the 1870s when a second wave of severe late blight was known to have occurred in Europe^38^. A plethora of reports on the cause and prevention were published during that time period. The Ngram search also revealed a peak in late blight mentions in the 1940s. Underlying data revealed that “*In 1946, Phytophthora infestans, without warning, caused an estimated $40,000,000 loss in tomatoes in the United States almost a century after the first reports in the US*^44^. Although the suddenness and extent of damage was severe in the US, the consequences were not as disastrous as nineteenth century disease in Ireland where potato was a sole food source for millions.

Contemporary surveillance of plant disease and subsequent deployment of management strategies requires up-to-date information about where disease is spreading. The immediacy of Tweets offers an opportunity for abundant, low latency data streams with extensive geographic data coverage, both within the U.S. and globally. Our Twitter data showed global engagement in conversation about late blight, but with the greatest volume of posts originating from the U.S. and Europe where late blight research and monitoring efforts are particularly active^17^. Refining posts to geolocated disease reports provided additional details useful to tracking the disease at the state level, and more occasional finer scale data (county, city). Both information extracted from text and locational data provided with Twitter posts offer potential new reports of the disease that might be missed by active surveillance. However, these methods still present challenges for disambiguating distinct locations from same-named places and manual revision was required to validate and improve the quality of data we produced. To analyze the errors, we evaluated specific cases where geocoding mis-identified geolocations in areas where late blight is not known to be present. For example, several counties in other US states were erroneously coded as same-named counties in large states like Texas when the correct state name was not included as context. Abbreviated place names at lower administrative levels without context (e.g., PA and VA for U.S. states Pennsylvania and Virginia) were geolocated as same-abbreviated countries (e.g., Panamá and Vatican City). In the future, custom-trained machine learning and NLP models could be developed to automatically and efficiently process disease observations from Twitter data sources and to support more concerted disease surveillance efforts like USABlight.org^30^ that rely on human reviewers to validate new data before it is mapped.

By leveraging large historic data records and more recent Twitter feeds for data analytics we have gained insight into both the epidemiological and societal impact of the first late blight pandemics in the US and better understand where modern global occurrences of the disease are now frequently reported. Extracting data from both of these voluminous sources required NLP techniques to identify themes of conversations and late blight reports containing geographic data. These sources presented certain distinct challenges (e.g., the spelling errors introduced by the physical condition of the historical documents and the URLs and hashtags in the social media posts required tailored preprocessing approaches) and opportunities (e.g., the prose form of the historical documents provides extensive context for each excerpt, while social media posts include metadata, such as geotags and user profile location, for extra context). Our work provides examples of how to overcome some of these challenges and leverage some of the opportunities. The tools we describe here will be open source and archived for future researchers interested in tracking and using social media and unstructured data as predictive intelligence for pandemic prevention.

## Methods

### Historical data source and preprocessing

We used scanned hard copies of the US Patent Reports from 1843 to 1845^25–27^ for this study. These documents were scanned with optical character recognition to convert the images of printed text to digital text and stored as Portable Document Format (PDF) files. To analyze the text, we first manually removed the tables, as there were few tables and automatic extraction did not handle these elements effectively. We converted the remaining content to ASCII text files using the Python PDFminer module (https://pdfminersix.readthedocs.io/en/latest/). Due to the age of the documents, some pages had incurred damage by the time they were scanned. As a result, the PDF files contained non-text artifacts, such as mildew stains, which appeared as extraneous marks in the digital version, causing spelling errors in the ASCII text files (e.g., ‘potato’ becomes ‘po;tato’). Additional misspellings were introduced by hyphens in words wrapped across two lines in the PDF (e.g., a line break between ‘pot-’ and ‘ato’ becomes ‘pot-ato’). To clean the files, we adapted Peter Norvig’s spell checking algorithm^45^. To correct misspellings, the algorithm weights probabilities of word selections based on a large body of text. We augmented the reference text to supply vocabulary suitable for our historical documents. The cleaned files were used as the corpora for word searches and natural language processing. In natural language processing, the term ‘corpus’ is used to refer to a collection of text to be analyzed. The report for each year was treated as an individual corpus. We used the built-in Python regular expression module (re) for key term searches. This process included searches for substring patterns within a string (e.g., searching “potato” should return “potatoes”) and removing false positive terms that match the query, but are not relevant in the intended context (e.g., to exclude “sweet potato” from a “potato” search result).

### Historical data text analysis

To effectively search the corpora, we identified key words and phrases that would indicate the presence of symptoms related to late blight. Since late blight had not been identified at the time of the reports’ publication, we used preliminary regular expression searches of potato within the documents to identify terms that were regularly associated with the presence of late blight in 1840’s vernacular, including “rot”, “murrain”, and “evil”. We also incorporated phrases indicative of symptoms, such as “decay” and “black spots”. We used the open-source Python Natural Language Toolkit library (NLTK)^46^ which applies statistical natural language processing to English language text. This library provides basic text processing tools such as tokenization (to break the corpus into parts such as words or sentences) and stemming (which supports finding variants of a word, such as “rot”, “rotting”, and “rotted”). We used NLTK to generate visual representations of the frequency and distribution of key terms within the corpus to explore co-occurrences of words, such as “potato” with “rot”.

We used NLTK and regular expressions to extract context windows around disease terms that were proximate to potato mentions in the corpora. To do this, we tokenized the corpus into sentences, selected target sentences containing disease terms and expanded the selection to include two sentences before and after the target sentence. If potato terms were also found within the 5-sentence context window, we collected this text for analysis, excluding any previously marked sentences.

### Historical data geoparsing

We geoparsed our selected potato disease context windows to develop a set of GIS coordinates associated with potential outbreaks. Geoparsing is the process of extracting place names from natural language text and assigning geographic coordinates to the location names. Extracting place names is performed using a natural language processing technique called Named Entity Recognition (NER), which labels words or phrases as entity types. For example, the labels may mark a word as ‘date’, ‘people’, ‘organization’, or ‘*geographic place*’. Geoparsing performs NER to get the text labeled by the NER as ‘geographic place’. Then the geographic places need to be assigned coordinates. Geoparsing relies on a gazetteer, a dictionary of place names and their coordinates. However, many places in different locations have the same name, so geoparsing uses a set of heuristics to assign geographic coordinates. We geoparsed our potato-disease text selections with CLAVIN-NERD^47^ connected to interactive web mapping interface, GazeGIS^48^. CLAVIN-NERD has been shown to perform well on formal texts, such as news and historical archives^49^. CLAVIN-NERD uses Standford NER, then applies a hierarchical set of rules to disambiguate the place names and select geographic coordinates defined within the GeoNames gazetteer (https://www.geonames.org/).

### Historical database setup and web map development

We created web maps with open-source platforms, PostGRESQL/PostGIS and GeoServer to manage and expose the geographic database and OpenLayers to display the spatial data as maps in a web browser (http://www.postgresql.org; http://postgis.net/; https://geoserver.org/; https://openlayers.org/). We imported results from GazeGIS/CLAVIN-NERD into a PostGIS relational database and used SQL commands to create a geometry column based on the coordinates of the data and the coordinate system World Geodetic System 1984 (WGS84) for publication by GeoServer.

The data for each year was treated as a separate layer in the interactive web map. The external JavaScript library *OpenLayers LayerSwitcher* was used to allow users to toggle between layers for each year on the same map. Map users could select a point to view the text from which it was generated. The interface also enabled users to enter metadata about each point and update the PostGIS database through the Web map.

### Historical data validation and spatial analysis

The data automatically extracted from the text required curation by subject experts to ensure that each point was relevant to the study question (i.e., Where was late blight located between 1843 and 1845?), was unique, and was correctly identified. Visualization on a map allowed subject experts to easily see if a point appeared out of place despite a correct location identification (e.g. “Oxford” referring to Oxford, England instead of Oxford, Massachusetts). To refine the results, we defined these five categories: accept (the information is relevant to the question and correctly located), move (the information is relevant but the location needs to be corrected), uncertain (the information might be relevant but requires further scrutiny by a second expert to confirm), archive (the information may be useful to adjacent questions (e.g. discussion of potential disease treatments) but has no relevant location), and remove (the information is irrelevant to the question). The Web map provided the capability to designate a category with a dropdown menu and add comments about each entry. The curated data (those points marked “accepted” or moved to the correct location and then accepted) were then imported into ArcGIS Pro ( https://www.esri.com/en-us/arcgis/products/arcgis-pro/overview) to produce georeferenced maps for late blight reports from 1843 to 1845.

### Google Ngram search

In addition to searching the Patent Office reports, we used Google Ngram Viewer^50^ to search for keywords in the Google Books collection for a broader view of the disease’s presence. For the purposes of the search, we focused on three terms: “potato disease”, “potato rot”, and “late blight”. The results were plotted as frequency distributions within texts published in the past 200 years.

### Twitter feed analytics

We queried Twitter posts (“Tweets”) between 2012 and 2022 from the commercial media aggregation service Brandwatch^40^ for regular expressions of all common and scientific names of *P. infestans* recorded in the EPPO GD (15 names, 7 languages, see Supplementary Table 2). This query returned 41,720 Tweets, of which 20,236 were novel posts (not “Retweets”). We relied on the post source location derived by Brandwatch^40^ to geolocate posts and mapped post intensity by country. Geolocation was available at the country scale for 22,879 Tweets (10,060 excluding Retweets). We relied on the post date to evaluate post intensity over time. The full results showed a high volume of posts in 2015 (12,126 Tweets, more than twice that of the next highest year, 2021 with 5355 Tweets). The majority of these Tweets (10,501 out of 12,126 Tweets) included mention of “EurekaMag”, a scholarly literature delivery service. We excluded these posts to focus our further analyses on the content shared on Twitter by a broader variety of users (11,570 Tweets over all 10 years). We further cleaned this data to remove URLs and eliminate duplicates, resulting in 9034 non-EurekaMag Tweets with unique text content.

#### Topic modeling with NLTK and LDA

To evaluate Tweet content, we generated two sets of topics derived statistically from word frequencies. We created an NLTK corpus using the text from all Tweets and evaluated 30 collocations (word pairs that appear together more often than expected by chance)^46^. This produced a set of initial topics in posts that included references to the Irish potato famine (“irish potato”, “potato famine”, “hambruna irlandesa”), potato-growing regions (“rift valley”), other plant, animal and human diseases (“bird flu”, “dengue fever”, “yellow fever”, “lassa fever”, “bacterial wilt”, “downy mildew”, “solani alternaria”, “early blight”, “ricin toxin”), disease pathways and research topics (“rxlr effectors”, “clonal lineages”, “resistance genes”, “technique accelerates”, “accelerates isolation”) and general terms pertaining to crop hosts, disease surveillance, and food security (“tomato plants”, “solanum tuberosum”, “potato variety”, “plant pathology”, “food security” “lutter contre”, “alerta temprana”). We used Latent Dirichlet Allocation (LDA) implemented with the Python package tomotopy to further model topics between documents (here, Tweets) in English (https://pypi.org/project/tomotopy/). LDA generates topics in a way that takes into account the common content found within documents, and each document is assigned a probabilistic distribution across the topics^51^. Each of 35 topics was described by its 10 most relevant words and its sum across document probability distributions (Supplementary Table 3).

We used keyword searches derived from this topic in the Twitter feeds to further evaluate direct observations of late blight. Manual evaluation of text revealed the presence of first-hand reports of the disease, frequently at specified geographic locations (e.g., a county, state, or country). Results for the terms “reported”, “confirmed”, “found in”, “found on”, “case of”, “spreading”, “identified” included descriptive geographic reports, primarily in locations in the US and Canada (e.g., “New Jersey tomato and potato crops threatened by late blight. 4 new cases reported in Salem County N.J. this week.”, “PEI agriculture officials now say late blight has been found in potato field in Freetown area”), as well as country-scale reports (e.g., “#Potato Late blight genotype EU_33_A2 reported in #Nigeria”) and several first-hand accounts of the disease (e.g., “Sad tonight about having to cut down all my tomato plants because of spreading late blight. Only 6 ripe fruit!”).

#### Text classification with Scikit-learn

To automatically identify Tweets that contain first-hand reports of the disease, we produced a hand-labeled dataset of positive and negative cases. We used unique posts in English from our topic modeling analysis, and machine translation to translate the remaining 2499 unique non-English Tweets. Translation was successful for 1750 Tweets, with rates of failure varying from 24 to 32% for Tweets in the top 5 non-English languages appearing in the dataset (Spanish, French, Romanian, German, Dutch). We randomly sampled 200 of the resulting 8665 Tweets and manually labeled each post as positive or negative for including direct observations or reports of late blight presence (termed “direct sighting”). Using the keywords previously identified with LDA, we labeled an additional 85 positive examples.

We used the labeled data set to train and evaluate several machine learning algorithms to automate classification of posts as “direct sightings” of late blight. For all methods, the original Tweet was cleaned to remove “at” tags (@ + a username), the Retweet flag (“RT”), and URLs. We used the Python Scikit-learn implementation of Term Frequency-Inverse Document Frequency (TF-IDF) to vectorize text, producing a matrix of unigram and bigram features for each post, ignoring English stopwords^52^. These features were used to train and evaluate four machine learning classification models: Linear Support Vector Classifier (Linear SVC), Logistic Regression, Decision Tree Classifier, and a Complement Naive Bayes Classifier with default parameters. K-fold cross validation (K = 10) was used to calculate average accuracy, precision, recall, and F-score metrics for each model, presented in Supplementary Table 4. Based on the mean F-score, which balances both Precision (the portion of positive predictions that are true positives) and Recall (the portion of true positives that are correctly predicted), we selected Linear SVC (mean F-score = 0.816, SD = 0.083) as the top performing model to apply to the remaining unlabeled posts. Using the Linear SVC model to label 8666 unique posts resulted in 1931 positive (posts containing information about a direct sighting of late blight) and 6735 negative posts (posts discussing other topics). The 10 most predictive unigram and bigram features were, in descending order by highest model coefficient: 'county', 'confirmed', 'tomato', 'reported', 'late blight', 'late', 'blight confirmed', 'blight', 'report', 'detected' for direct sighting, and 'menace', 'menace explained', 'variety', 'pathogen phytophthora', 'new', 'virus', 'plant', 'early', 'resistant', 'resistance' for posts on other topics. Code from model development and evaluation is available in the online supplementary materials.

#### Geoparsing with named entity recognition (NER) and OpenStreetMap

We adapted the approach used by Karimzadeh^41^ to geoparse (i.e., recognize and geolocate) place names mentioned in Tweets classified as positive reports for late blight. We used the spaCy English Core Web Medium (en_core_web_med) pipeline and pre-trained model to identify locations and geopolitical entities in on-topic posts for Named Entity Recognition and the OpenStreetMap (OSM) geocoder to geolocate the resulting named places (https://spacy.io/; https://geocoder.readthedocs.io/providers/OpenStreetMap.html). OSM provides ranked results of same-named places derived from Wikipedia importance ranking. To further disambiguate between same-named places (“toponym resolution”), we implemented a series of heuristics to integrate additional spatial context from the Tweet, in order of preference: (1) *separate feature code boosting pipelines for abbreviations*: two- and three-character place names (typically state or country abbreviations) were geocoded with no additional context, (2) *exact match query on the “name” field of toponyms*: exact matches for countries, continents, and regional groupings were geocoded with no additional context, (3) *hierarchical disambiguation constrained to consecutive names*: adjacent place names in posts were evaluated for a hierarchical relationship (e.g., geo-entities “Long Island” and “NY” geocoded from “Long Island, NY” when the entities appeared within 4 characters of each other in a post) and (4) if a Tweet’s origin country is provided, prefer locations within that country. This produced a data point for each location mentioned at the country, state, or city scale and geographic coordinates. Two authors manually evaluated all positive reports and locations for both classification and geoparsing tasks. False positive reports (incorrect classification) were excluded. Cases where geoparsing produced incorrect locations or missed locations were replaced with corrected or missed place names that were geocoded as above. We aggregated this validated data to the country scale to produce a global *P. infestans* map, and to the state and city scale to produce a map of *P. infestans* reports in the United States and Canada.

### Supplementary Information

Supplementary Information.

## Data Availability

The datasets generated and/or analysed during the current study are available in the Github repository, https://github.com/lgtateos/late_blight_text_mining.
