# Supplementary material for: Reconstructing historic and modern potato late blight outbreaks using text analytics
Source: Sci Rep. 2024 Feb 15;14:2523. doi: 10.1038/s41598-024-52870-2 (PMC10869797; doi:10.1038/s41598-024-52870-2)
Supplement: Supplementary file 1 — Supplementary Information. [file 41598_2024_52870_MOESM1_ESM.docx]

**Supplementary Materials for**

**Title:** Reconstructing Historic and Modern Potato Late Blight Outbreaks using Text Analytics

**Authors**

Ariel Saffer^1^, Laura Tateosian^1^ , Yi-Peng Yang^1^, Amanda Saville^2^, and Jean B Ristaino^2,3^*,

**Affiliations**

^1^Center for Geospatial Analytics, NC State University, Raleigh, NC

^2^Department of Entomology and Plant Pathology, NC State University, Raleigh, NC

^3^Emerging Plant Disease and Global Food Security Cluster, NC State University, Raleigh, NC

*Corresponding Author: Jean B Ristaino Jean_ristaino@ncsu.edu

**
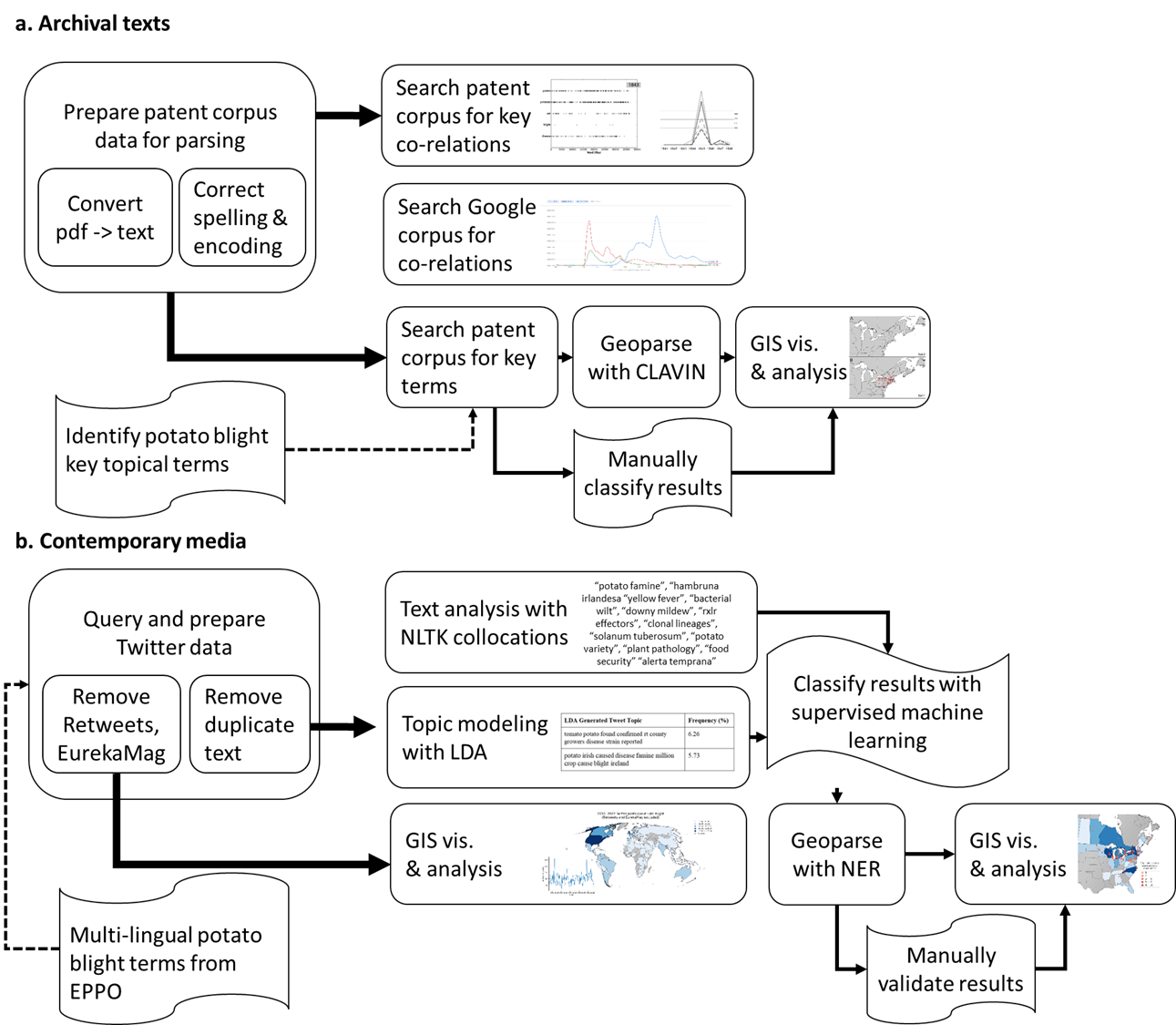
**

**Supplementary Figure 1.** Schematic of the workflow established to convert unstructured text data from US Commissioner of Patent reports (1843-45) and Tweets (2011-2021) to create maps for visualization of location information over time.

**Supplementary Table 1**. Glossary of text analytics terms and tools referenced throughout the paper.

| **Term** | **Plain-language description** |
| --- | --- |
| NLP (Natural Language Processing) | A field of artificial intelligence that focuses on the interaction between computers and humans using natural language. |
| NLTK (Natural Language Toolkit) | A Python library for working with human language data, providing tools for tasks like tokenization, stemming, tagging, parsing, and more. |
| Topic Modeling | A technique in text mining and NLP that identifies topics present in a corpus of text documents. |
| LDA (Latent Dirichlet Allocation) | A probabilistic method used for topic modeling, which assumes that documents are mixtures of topics and topics are mixtures of words. |
| Text Classification | The process of assigning predefined categories or labels to text based on its content. |
| TF-IDF (Term Frequency-Inverse Document Frequency) | A statistic that reflects the importance of a word in a document relative to a collection of documents, used to represent text as numbers. |
| Collocation | The habitual juxtaposition of a particular word with another word or words with a frequency greater than chance. |
| Machine Learning Classification Models | Statistical models trained to classify input data into predefined categories.  Examples of traditional machine learning models include Linear SVC, Decision Tree Classifier, Complement Naive Bayes Classifier |
| *Linear SVC (Support Vector Classification)* | A type of support vector machine (SVM) algorithm used for binary and multiclass classification. |
| *Decision Tree Classifier* | A machine learning algorithm that makes decisions by recursively splitting the dataset into subsets based on the most significant attributes. |
| *Complement Naive Bayes Classifier* | A variant of the Naive Bayes classifier that is particularly effective for imbalanced text classification tasks. |
| Geoparsing | The process of extracting and identifying geographic information from text data. |
| CLAVIN Geoparser | An open-source geoparsing tool that extracts location information from unstructured text. |
| Google Ngram | A service that analyzes the frequency of words or phrases in a large collection of books and documents over a specified period. |
| Named Entity Recognition (NER) | The process of identifying and classifying named entities (e.g., persons, organizations, locations) in text. |
| spaCy | An open-source Python library for advanced natural language processing tasks, providing pre-trained models and tools for tokenization, POS tagging, and other tasks. |
| Sci-kit Learn (or scikit-learn) | A machine learning library in Python that provides simple and efficient tools for data analysis and modeling, including text analytics. |
| PostGRESQL/PostGIS | A database with a Geographic Information System (GIS) extension for managing and analyzing spatial data. These tools are not specifically designed for text analytics but can be used in conjunction with location-based text data. |
| Open Layers | An open-source JavaScript library used for displaying dynamic maps on web pages |

**Supplementary Table 2.** Common names of *P. infestans* in multiple languages, extracted from the European and Mediterranean Plant Protection Organization (EPPO) Global Database.

| **Common Name** | **Language** |
| --- | --- |
| kartoffelskimmel | Danish |
| downy mildew of potato | English |
| late blight of potato | English |
| late blight of tomato | English |
| mildiou de la pomme de terre | French |
| mildiou de la tomate | French |
| Braunfäule: Kartoffel | German |
| Braunfäule: Tomate | German |
| Kraut- und Knollenfäule: Kartoffel | German |
| Krautfäule: Tomate | German |
| bulvių maras | Lithuanian |
| фитофтороз картофеля | Russian |
| mildiú de la patata | Spanish |
| mildiú del tomate | Spanish |
| tizón tardio | Spanish |

**Supplementary Table 3**. Topics in Tweets about *P. infestans* modeled with Latent Dirichlet Allocation (LDA)^1^.

| LDA Generated Tweet Topic | Frequency (%) |
| --- | --- |
| tomato potato found confirmed rt county growers disease strain reported | 6.26 |
| potato irish caused disease famine million crop cause blight ireland | 5.73 |
| potato disease potatoes resistant farmers crops help crop could new | 4.69 |
| tomato early blight good leaf weather late tomatoes diseases conditions | 4.34 |
| potato resistant variety farmers disease new scientists developed yields smallholder | 4.14 |
| effector rxlr host plant potato targets via avr3a immune effectors | 3.51 |
| potato resistance gene new gm solanum r molecular genes found | 3.51 |
| resistance potato genes new wild isolation accelerates technique varieties species | 3.46 |
| potato tomato diseases detected threat global still rt food plant | 3.42 |
| potato pathogen population disease new center study structure populations talk | 3.41 |
| would new virus great time know also get one years | 3.37 |
| potato tomato like growing plants get one crop look last | 3.37 |
| potato resistant control varieties use fungicide fungicides potatoes developed dutch | 3.23 |
| tomato early growers treatment organic year seed using fungicide take | 3.12 |
| tomato plants potato devastating united eastern states disease rt threatens | 2.87 |
| potato resistance pathogen genes gene oomycete plant recognition detection novel | 2.78 |
| potato irish famine rt lineage fall triggered rise pathogen strain | 2.7 |
| potato plant genetically engineered online read pathology disease latest usda | 2.64 |
| pathogen gene expression molecular new looking plant silencing paper growth | 2.36 |
| potato tomato resistance breeding using fight use plants work john | 2.33 |
| potato control spread new crop caused oomycete disease rapidly reduced | 2.25 |
| shows potato field resistance first gm extreme phytophthora observation managing | 2.2 |
| potato managing oak tips sudden death particularly field long risk | 2.19 |
| potato phd effectors network haustoria europe work evolution studies ireland | 2.13 |
| new potato cell resistance actin rt formation wall focus crops | 2.09 |
| rt de analysis tomato agriculture talking plants blue et canada | 2.07 |
| next tomato plants gene potato garden using hope year michigan | 2.03 |
| clonal potato tomatoes biggest lineages resistance probably growing within new | 2.03 |
| potato new could variety farmers east spore protein control blight | 1.96 |
| potato disease serious farmers agriculture well education students privatization longer | 1.85 |
| potato photo news major trafficking vesicle proteins rapidly rxlr throughout | 1.8 |
| host de metabolic two interaction effectors rt range nice analysis | 1.65 |
| potato model scientist wins teen nyc math new blight student | 1.61 |
| downy mildew life different isolates next mefenoxam used diversity stages | 1.54 |
| yellow valley newcastle rift us fever lassa dengue bird eee | 1.34 |

^1^ LDA is a generative statistical technique that models a set of probable topics across a series of texts using word frequency within and between documents (Blei, Ng, & Jordan, 2003). In this table, each topic is defined by its 10 most probable words as they appear in the original Tweets.

**Supplementary Table 4.** Performance metrics^2^ of Machine Learning models evaluated for text classification.

|  | Average Metric (Mean ± Standard Deviation) | | | |
| --- | --- | --- | --- | --- |
| Machine Learning Model | Accuracy | Precision | Recall | F-score |
| Linear SVC | **0.863 ±0.04** | 0.839 ±0.061 | 0.795 ±0.099 | **0.812 ±0.064** |
| Logistic Regression | 0.818 ±0.034 | **0.89 ±0.085** | 0.591 ±0.103 | 0.705 ±0.092 |
| Decision Tree Classifier | 0.677 ±0.098 | 0.582 ±0.147 | 0.702 ±0.18 | 0.619 ±0.117 |
| Complement Naive Bayes | 0.842 ±0.054 | 0.782 ±0.109 | **0.814 ±0.078** | 0.794 ±0.081 |

^2^ Metrics are averaged across 10 training and test data splits. The top performing model by each metric is indicated in bold.
